# The Acute Effect of Hydroxychloroquine Sulfate on Hunger, the Plasma Concentration of Orexigenic Peptides and Hedonic Food Intake: A Pilot Study

**DOI:** 10.3390/nu15194264

**Published:** 2023-10-05

**Authors:** Emily Ruilova Sosoranga, Wout Verbeure, Hannelore Geysen, Theo Thijs, Christophe Matthys, Inge Depoortere, Jan Tack

**Affiliations:** 1 Translational Research Center for Gastrointestinal Disorders, KU Leuven, 3000 Leuven, Belgiuminge.depoortere@kuleuven.be (I.D.); 2 Clinical and Experimental Endocrinology, KU Leuven, 3000 Leuven, Belgium; christophe.matthys@uzleuven.be; 3 Department of Endocrinology, University Hospitals Leuven, 3000 Leuven, Belgium; 4 Department of Gastroenterology and Hepatology, University Hospitals Leuven, 3000 Leuven, Belgium

**Keywords:** bitter, appetite, ghrelin, motilin, fasting, Plaquenil^®^, hydroxychloroquine sulfate, quinine

## Abstract

The direct infusion of bitter solutions in the gastrointestinal tract can reduce the secretion of orexigenic hormones and influence appetite and food intake. We aimed to explore whether oral ingestion of the bitter tastant hydroxychloroquine sulfate can exert similar effects. Ten lean adult women were included in this double-blind, randomized, two-visit, crossover study. After an overnight fast, each volunteer received film-coated tablets containing 400 mg of hydroxychloroquine sulfate (Plaquenil^®^) or placebo. Plasma-ghrelin, -motilin, -insulin and blood-glucose concentrations were determined every 10 min before and 30 min after feeding; appetite was scored every 10 min. Hunger scores were investigated with a special interest 50–60 min after the ingestion of hydroxychloroquine sulfate, right before a rewarding chocolate milkshake was offered to drink ad libitum. Compared with the placebo, hydroxychloroquine sulfate tended to reduce hunger at the time of interest (*p* = 0.10). No effect was found upon subsequent milkshake intake. Motilin plasma concentrations were unaltered, but acyl-ghrelin plasma concentrations decreased after the ingestion of hydroxychloroquine sulfate (t = 40–50; *p* < 0.05). These data suggest that the oral intake of hydroxychloroquine sulfate tablets reduces subjective hunger via a ghrelin-dependent mechanism but does not affect motilin release, hedonic food intake or insulin levels in healthy women.

## 1. Introduction

Bitter flavors are frequently associated with spoiled or toxic substances [1]. As a result, the lingual detection of bitterness through taste 2 receptors (TAS2Rs) elicits an aversive response, reducing the intake of potentially noxious agents [2,3]. TAS2Rs are expressed in several other tissues throughout the body, including in enteroendocrine cells (EECs), goblet cells and the Paneth cells of the gastrointestinal (GI) tract [4,5,6,7,8]. These TAS2Rs detect bitter compounds in the lumen derived from ingested food or medical drugs or released by bacteria to elicit an appropriate functional response. To limit further intoxication, bitter agents appear to trigger a repulsive reaction in the GI tract as well, manifested in a delay in gastric emptying, decreased accommodation and early cessation of food intake [9,10,11]. In addition, signaling through TAS2Rs that are located on gastric smooth muscle cells may reinforce the repulsive effect by directly affecting GI motility [9]. 

The reaction caused by bitter tastants in the GI tract is, at least in part, linked to the altered secretion of appetite-regulating hormones. Both in vivo studies on animals and humans and in vitro studies on cell lines and human primary gastric crypt preparations have shown that bitter agonists can affect the secretion of appetite-regulating hormones [10,12,13,14,15,16]. These hormones can be subdivided into two groups: the anorexigenic or appetite-suppressing hormones and the orexigenic or appetite-stimulating hormones to which acyl-ghrelin and motilin belong. Acyl-ghrelin is the acylated, active form of the gut peptide ghrelin, which is primarily released in the stomach by P/D1 cells and stimulates food intake [17,18,19]. Motilin is produced by M-cells, a subtype of EECs located mainly in the proximal part of the duodenum [20]. Motilin fulfills a well-established role in stomach motility during the interdigestive state [21]. Additionally, increases in motilin plasma concentration correlate with intense hunger bursts, highlighting the orexigenic role of motilin [22]. A recent study demonstrated that the intragastric administration of the bitter compound quinine hydrochloride (QHCl) via a nasogastric feeding tube in healthy volunteers significantly reduced the plasma motilin concentration, which seemed to be associated with a reductive effect on hunger sensations [10]. Additionally, brain imaging studies of healthy volunteers showed that the intragastric administration of QHCl altered brain activity in homeostatic and hedonic regions that covaried with QHCl-induced decreases in circulating levels of acyl-ghrelin and motilin. In the same study, both prospective and hedonic food intake were reduced [16]. These findings underline the potential of the targeted delivery of bitter agents in the stomach for weight-loss treatment. Moreover, bitter agents might also be beneficial in patients with obesity-related type 2 diabetes, considering that intragastric and intraduodenal QHCl administration in the fasting state or after a mixed nutrient drink elevated insulin plasma values, resulting in lower glucose levels in healthy volunteers [13,23]. 

Although the direct infusion of bitter solutions in the GI tract shows promising effects, the expansion of its use is hampered by the need for nasogastric intubation. Replacement with a so-called “bitter pill” that releases its content in the stomach could offer a solution to overcome this barrier. Such a pill is already commercially available under the name Plaquenil^®^. It has anti-parasitic and anti-inflammatory properties used to prevent and treat malaria and to treat autoimmune diseases [24]. The main component, hydroxychloroquine sulfate (HCQS), is a synthetic relative of chloroquine [25]. HCQS, chloroquine and QHCl share a similar quinoline ring structure [26], which determines the bitter taste. 

This study aims to investigate whether the intake of a pill comprising HCQS can have similar effects on hunger sensations, hedonic food intake and orexigenic gut hormone release compared with previously reported [10,27] intra-gastric QHCl administration in healthy, lean women. Additionally, HCQS is known to promote a hypoglycemic effect by stimulating insulin release [28,29]. Therefore, the effects on blood glucose and insulin plasma concentrations were investigated as well. This information may open the road to investigate whether a “bitter pill” may be useful as an add-on therapy for the treatment of obesity and obesity-related type 2 diabetes.

## 2. Materials and Methods

### 2.1. Ethical Approval 

The trial was approved by the Leuven University Hospital Medical Ethics Committee (S61870 11/12/2018) and registered at clinicaltrials.gov as NCT04005768. All study procedures were performed in full accordance with the Declaration of Helsinki. All participants approved and signed the informed consent before being included in the study and were allowed to withdraw at any time for any reason.

### 2.2. Study Population and Sample Size

**Figure 1:**
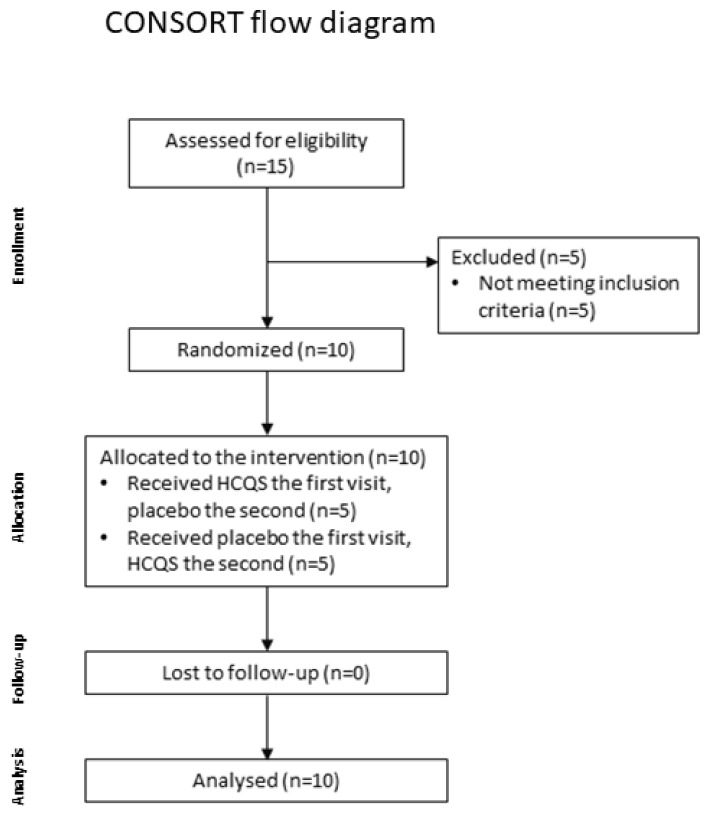
CONSORT flow diagram depicting the progression of participants through the study. Ten eligible participants were randomly assigned according to a computer-generated allocation schedule to 1 of 2 treatment sequences: hydroxychloroquine sulfate (HCQS) the first study visit and placebo the second study visit or the placebo the first study visit and HCQS the second study visit. All randomized participants completed the study.

Ten healthy lean female volunteers between the ages of 18 and 65 were included in this proof-of-concept study (Figure 1). Their BMIs were situated between 18 and 25 kg/m^2^. The volunteers were not dieting and had a stable body weight for at least 3 months. In order not to exceed the maximal dose of 6.5 mg HCQS per kg body weight after the intake of two tablets of Plaquenil^®^, the volunteers needed to have a minimal body weight of 62 kg. Volunteers needed to use a highly effective method of birth control. Finally, volunteers had to score 60 or higher on a 100 mm visual analogue scale (VAS) for liking a chocolate milkshake to ensure its rewarding nature. Potential subjects were excluded if they met any of the following criteria: history of GI or other significant somatic or psychiatric diseases; known drug allergies; diabetes; significant heart, lung, liver or kidney diseases; a prolonged QT-interval > 450 ms or other symptoms that HCQS can worsen during long-term use; food allergies or lactose intolerance. 

As reported in a previous study [16], fourteen female volunteers are sufficient to obtain a quinine effect on motilin release with 80% power and α = 0.05. These trends were already clear in ten volunteers in a previous study [9]. We, therefore, opted to recruit ten healthy volunteers for this pilot study to confirm the equal performance of the Plaquenil^®^ tablet compared to a previously reported study where a QHCL solution was administered in the stomach [9]. Only female volunteers were included as they exhibit a higher degree of GI sensitivity for bitter compounds [11,30]. 

### 2.3. Test Compounds

Participants received two tablets of Plaquenil^®^ (Sanofi-Synthelabo Limited, Newcastle, UK) or two placebo tablets (Laboratoria Wolfs, Zwijndrecht, Belgium), administered in a double-blinded, crossover fashion. In total, the two Plaquenil^®^ tablets contained 400 mg of HCQS, which equals 310 mg of hydroxychloroquine (HCQ). 

### 2.4. Study Design and Protocol 

During the screening visit, the study protocol was explained; the informed consent form was signed; and in- and exclusion criteria were checked. Weight, height, waist–hip ratio and body composition were measured. The latter was determined using a Bodystat1500 (Bodystat Ltd., Douglas, Isle of Man). Subjects tasted the chocolate milkshake for “liking” and were only included when they rated a score of 60 or higher on a 100 mm VAS to ensure the rewarding nature of the milkshake. An electrocardiography (ECG) confirmed the absence of a prolonged QT interval. Volunteers filled in the online screening questionnaires, including the Dutch version of the Rome IV criteria questionnaire to confirm the absence of functional GI disorders. 

**Figure 2:**
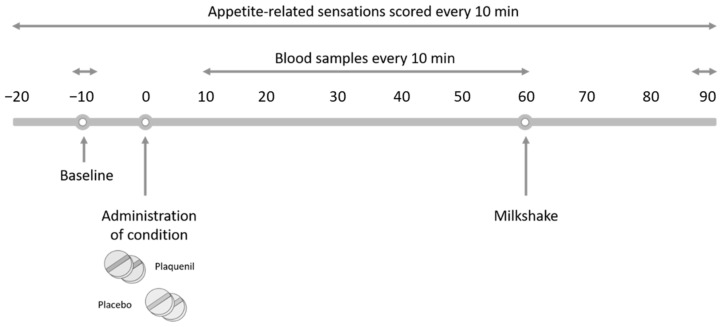
Schematic overview of the study design. First, an intravenous dwelling line was positioned for blood sample collection. A baseline blood sample was taken at timepoint −10. At timepoint 0, two tablets of Plaquenil^®^ (HCQS, 400 mg) or two tablets of placebo were administered. For a period of 1 h, blood samples were collected every 10 min. At timepoint 60, volunteers were offered a chocolate milkshake from which they could drink until they felt fully satisfied. A last blood sample was taken at timepoint 90 to estimate gut peptide recovery. At every timepoint presented in the schematic overview (from −20 until 90), the volunteer scored appetite-related sensations on a 100 mm visual analog scale.

This study was a placebo-controlled, double-blind, randomized, two-visit crossover study (Figure 2). Volunteers were requested to have a simple bread meal the evening before their visit. After an overnight fast of at least 12 h, the volunteers arrived at the hospital. An intravenous catheter was placed and continuously perfused with saline during the entire study visit. After a 10 min rest period, a baseline blood sample was taken (time = −10) for the measurement of motilin, acyl-ghrelin, insulin and blood glucose. After ten minutes, two tablets of Plaquenil^®^ (total concentration 400 mg HCQS) or placebo were administered orally with 240 mL of water (time = 0). Then, blood samples were collected for one hour, every 10 min. Thereafter, volunteers were instructed to a drink chocolate milkshake ad libitum until they felt fully satisfied (time = 60). After 30 min, the last blood sample was taken and volunteers returned home (time = 90). For the duration of the whole study, volunteers were requested to score appetite-related sensations (hunger, prospective food intake and satiety) and symptoms (belching, nausea, fullness, cramps and pain) every 10 min on a 100 mm VAS. Hunger was rated as “How hungry do you feel?”, prospective food intake as “How much do you think you can eat?” and satiety as “How satisfied do you feel?”. A rating of 0 mm meant “Not at all”, and 100 mm meant “Very much”. A 2-week washout period between the study visits was respected to avoid any potential carryover effects. 

### 2.5. Hedonic Food Intake Measurement 

Before each study visit, a fresh chocolate milkshake was prepared by combining 8 scoops of vanilla ice cream (Ijsboerke, Tielen, Belgium), 710 mL of 2% milk (Lactel, Brussels, Belgium) and 4 tablespoons of Imperial’s Chocolate Topping (Continental Foods, Puurs, Belgium). Volunteers drank from a 200 mL beaker that was repeatedly refilled with chocolate milkshake until the volunteers expressed that they felt comfortably full. The milkshake was weighed before and after ad libitum consumption, taking into account that each ingested gram of chocolate milkshake equaled 1 kcal. This approach to measuring hedonic food intake is based on a publication from Iven et al. [16], who adapted their work from Stice et al. to Belgian brands [31]. 

### 2.6. Blood Collection

Blood samples for hormone analysis were collected in lithium–heparin-coated tubes (Becton, Dickinson and Company, Franklin Lakes, NJ, USA) for motilin analysis and ethylenediaminetetraacetic acid (EDTA)-coated tubes (Becton, Dickinson and Company, Franklin Lakes, NJ, USA) for ghrelin and insulin analysis. Blood was supplemented with 500 kIU aprotinin/mL of blood. Blood collected for ghrelin measurements was additionally supplemented with 0.57 mM of phenylmethylsulfonyl fluoride solution (Sigma-Aldrich, Steinheim, Germany). Samples were centrifuged at 4 °C for 10 min at 1400× *g*, after which, plasma was collected. Plasma for acyl-ghrelin was further acidified to a final concentration of 0.1 N HCl, extracted using a Sep-Pak C18 column (Waters Corporation, Milford, MA, USA) and vacuum-dried. All samples were stored at −80 °C until further analysis. Insulin concentration was measured using the EK-035-06 kit (Phoenix Pharmaceuticals, Burlingame, CA, USA), while motilin and acyl-ghrelin were measured using an in-house radio-immuno-assay (RIA), as described before [32,33]. 

### 2.7. Statistical Analysis

For the statistical analysis, the SAS University Edition software v9.4 (SAS Institute, Cary, NC, USA) was used. To ensure that all the assumptions were met, logarithmic boxcox transformations were applied if data were not normally distributed. These data were presented as the transformed values. Significance was set at *p* < 0.05, and the data were presented as mean ± SEM. First, baseline values (t = −10) were compared between conditions using a two-tailed paired *t*-test. Because no significant baseline differences between conditions were found, appetite-related sensations (hunger, prospective food intake and satiety), gut hormone concentrations (insulin) and blood glucose levels were analyzed as absolute changes from baseline (∆). For appetite-related sensations and blood glucose levels, the effect of treatment on these ∆variables over time was analyzed using marginal linear mixed models for the fasted (t = 10–60) and fed state (t = 70–90 for appetite-related sensations and t = 90 for hormones and glucose) separately. For these variables and for plasma hormone concentrations, a planned contrast analysis was performed to see if there was a difference between treatments 50–60 min after administration. ANCOVA mixed models were performed to better account for the effect of treatment over time on orexigenic hormone concentrations (motilin, acyl-ghrelin) and the correlation of gut peptide concentrations within patients. The “time” and “treatment by time interaction” effects were set as linear and quadratic covariance parameters. The treatments (HCSQ, placebo) and distinct study phases (baseline, t = −10; fasted state, t = 10–60; fed state, t = 90) were included as fixed effects. To account for the correlation between the timepoints in each volunteer, time and treatment were treated as random effects with the patient set as the random variable with an autoregressive order 1 (AR1) covariance structure specified. To state the model’s degrees of freedom, the Satthertwaite method was chosen. LRT tests were performed to test the linear and quadratic covariance parameters “time” and “treatment by time interaction” for significance at the 5% level. Milkshake intake and taste were analyzed using a one-tailed paired *t*-test. Symptom scores below a cut-off of 10 out of 100 mm VAS were not considered in this study design. The area under the curve (AUC) of symptom severity above the 10 mm cut-off was calculated for the fasted and fed states separately and compared between the placebo and HCQS condition with a Wilcoxon matched-pairs test. Data are presented as interquartile range (IQR). 

## 3. Results

### 3.1. Demographics

Fifteen volunteers signed the informed consent form. Five were excluded because of a rating lower than 60 out of 100 for liking the milkshake or because they did not meet other inclusion criteria. Ten volunteers (BMI of 22.8 ± 0.3 kg/m^2^; age 24 ± 1 years) were included in the study. These volunteers had a waist-hip ratio of 0.73 ± 0.01; a fat percentage of 23.4 ± 0.9%; a lean mass of 51 ± 1 kg; a dry lean mass of 16 ± 0.4 kg; and a water percentage of 53 ± 0.7%.

### 3.2. Appetite-Related Sensations

**Figure 3:**
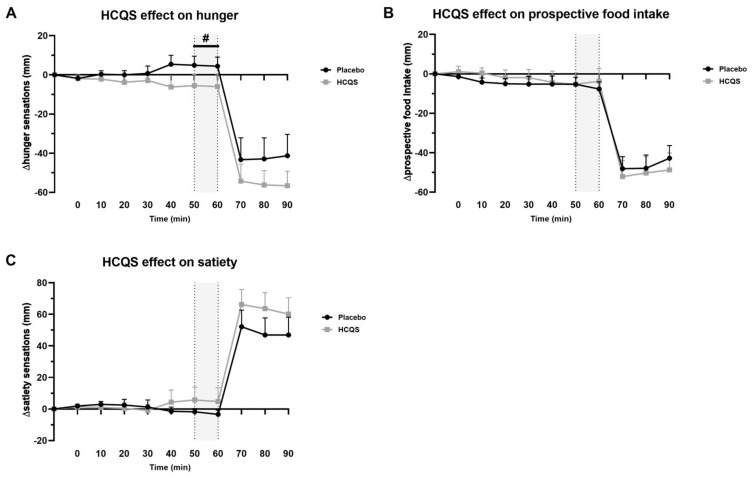
Hydroxychloroquine sulfate tends to reduce hunger ratings 50–60 min after ingestion. Effect of placebo and hydroxychloroquine sulfate (HCQS, 400 mg) on (**A**) hunger sensations, (**B**) prospective food intake and (**C**) satiety sensations (*n* = 10). Data are presented as absolute values adjusted by baseline (time = −10) and presented as mean ± SE. Placebo or HCQS tablets were taken at timepoint 0, and after 60 min, volunteers drank ad libitum from a rewarding chocolate milkshake. An a priori treatment effect was expected 50 to 60 min after intake (gray area), which was analyzed with a planned contrast analysis in a marginal linear mixed model (# Padj = 0.10).

Mean changes from baseline (∆) in VAS ratings for appetite-related sensations (hunger, prospective food intake and satiety) are shown in Figure 3. Absolute baseline hunger sensations for the placebo (63 ± 6 mm) and HCQS (70 ± 6 mm) treatment did not differ significantly (*p* = 0.42). After placebo administration, hunger increased by 5 ± 3 mm; conversely, a reduction of −6 ± 4 mm was found after the HCQS treatment. The ∆change in hunger scores from baseline was maximal after 50–60 min but did not reach statistical significance (*p* = 0.10; Figure 3A). After the consumption of the chocolate milkshake, hunger scores for both conditions decreased (*p* < 0.0001) without a difference between conditions (*p* = 0.35). 

Baseline values of prospective food intake were 69 ± 2 mm for the placebo group and 66 ± 7 mm for the HCQS group (*p* = 0.67). Prospective food intake values decreased by −7 ± 3 mm from baseline for the placebo and −5 ± 4 mm for HCQS at the time of interest (Figure 3B). The decrease was not significantly different between conditions (*p* = 0.53). Drinking the milkshake strongly affected the volunteer’s prospect of food intake (*p* < 0.0001), but this was not condition-dependent (*p* = 0.70). 

Absolute satiety baseline values were not normally distributed and transformed to 2.5 ± 0.4 mm for the placebo and 1.6 ± 0.4 mm for HCQS, which showed no significant difference (*p* = 0.11). The original baseline scores were 19 ± 7 mm for the placebo and 12 ± 7 mm for HCQS. The satiety scores increased by 5 ± 6 mm from baseline 50–60 min after the administration of HCQS and decreased by −3 ± 2 mm after placebo administration (*p* = 0.18) (Figure 3C). However, this difference in satiety scores did not reach statistical significance in our pilot sample. Satiety increased strongly after milkshake consumption (*p* < 0.0001), but this increase was similar across conditions (*p* = 0.33). 

### 3.3. Symptoms

The results of symptoms (belching, nausea, fullness, cramps and pain) reported during the study visits are presented in Table 1. The *p*-values were only obtained for the fullness symptom, which was the only non-zero comparison. No significant differences between treatments were observed in fullness in the fasted state (*p* > 0.99) or the fed state (*p* = 0.20). 

### 3.4. Orexigenic Gut Peptides

**Figure 4:**
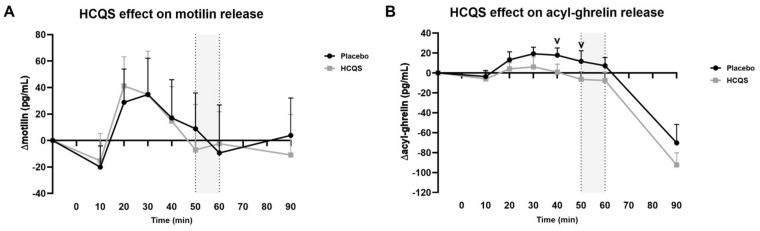
Hydroxychloroquine sulfate reduces acyl-ghrelin plasma concentrations 40–50 min after ingestion. Effect of placebo and hydroxychloroquine sulfate (HCQS, 400 mg) on (**A**) motilin plasma concentrations and (**B**) acyl-ghrelin plasma concentrations (*n* = 10). Data are presented as absolute values adjusted by baseline (time = −10) and presented as mean ± SE. Placebo or HCQS tablets were taken at timepoint 0, and after 60 min, volunteers drank ad libitum from a chocolate milkshake. The gray area marks the timepoint where the treatment effect was expected a priori. Statistical analysis with ANCOVA mixed models showed an HCQS treatment effect on acyl-ghrelin plasma concentrations at timepoint 40 and 50 v, *p* < 0.05 (not corrected for multiple comparisons). The planned contrast analysis was performed with marginal linear mixed models but showed no statistical significance at that timepoint (Padj = 0.11).

Absolute motilin plasma levels did not differ significantly between conditions at baseline (+887 ± 30 pg/mL for the placebo and 845 ± 27 pg/mL for HCQS). For both conditions, the motilin plasma concentration fluctuated over time during the fasted state (*p* < 0.001; Figure 4A). There was no significant difference in the ∆motilin plasma concentration between the two treatments, not even at the anticipated timepoint of 50–60 min after administration (0 ± 22 pg/mL for the placebo and −5 ± 20 pg/mL for HCQS). Similarly, the concentration of the last motilin plasma sample, 30 min after the ingestion of the chocolate milkshake, was not significantly different between conditions (4 ± 28 pg/mL for the placebo and −11 ± 31 pg/mL for HCQS).

The baseline values of acyl-ghrelin were not significantly different between conditions, with concentrations of 171 ± 13 pg/mL and 178 ± 14 pg/mL for the placebo and HCQS, respectively. Overall, the ∆acyl-ghrelin values tended to be lower after the ingestion of HCQS compared with the placebo (Figure 4B). ANCOVA mixed models analysis showed a statistically significant difference between treatments 40 and 50 min after the administration of HCQS (*p* < 0.05), which was lost after correcting for multiple comparisons. At the prespecified timepoint 50–60 min after ingestion, the average ∆acyl-ghrelin values were 9 ± 7 pg/mL for the placebo and −7 ± 6 pg/mL for HCQS (*p* = 0.11). Thirty minutes after drinking the milkshake, acyl-ghrelin concentrations dropped to −70 ± 18 pg/mL for the placebo and −92 ± 12 pg/mL for HCQS, which was not significantly different.

### 3.5. Effect on Insulin Release and Blood Glucose Levels

**Figure 5:**
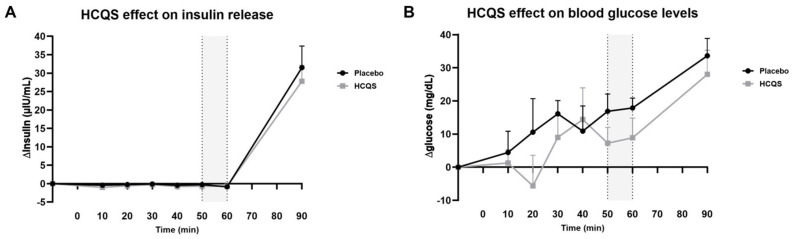
Hydroxychloroquine sulfate does not appear to influence plasma insulin concentrations or glucose levels. Effect of placebo and hydroxychloroquine sulfate (HCQS, 400 mg) on (**A**) insulin plasma concentrations and (**B**) blood glucose levels (*n* = 10). Data are presented as absolute values adjusted by baseline (time = −10) and presented as mean ± SE. Placebo or HCQS tablets were taken at timepoint 0, and after 60 min, volunteers drank ad libitum from a chocolate milkshake. The gray area marks the timepoint where the treatment effect was expected a priori. Statistical analysis was performed with marginal linear mixed models.

The absolute insulin baseline values for the placebo and HCQS were 2.3 ± 1.0 and 2.2 ± 1.1 µIU/mL, respectively (Figure 5). These data were not normally distributed. After transformation, there was no significant difference found between treatments (0.51 ± 0.19 µIU/mL for the placebo and 0.42 ± 0.19 µIU/mL for HCQS). Fasted insulin concentrations decreased by −0.5 ± 0.4 µIU/mL from baseline for the placebo and −0.7 ± 0.3 µIU/mL for HCQS (Figure 5A), which was not significantly different between treatments 50–60 min after administration (*p* = 0.75). ∆Insulin plasma concentrations increased strongly after chocolate milkshake intake by 31.5 ± 5.9 µIU/mL for the placebo and 27.8 ± 4.3 µIU/mL for HCQS, but the difference was also not significant. 

Whole-blood glucose levels were not significantly different at baseline (68 ± 5 mg/dL for the placebo and 66 ± 7 mg/dL for HCQS; *p* = 0.59). Glucose values increased by 17 ± 3 mg/dL from baseline for the placebo and by 8 ± 4 mg/dL for HCQS 50–60 min after administration (Figure 5B), which was not significantly different (*p* = 0.32). Similarly, glucose levels did not differ significantly between treatments 30 min after milkshake consumption. 

### 3.6. Chocolate Milkshake Intake

The volume intake of the rewarding chocolate milkshake did not differ significantly (*p* = 0.20) after the intake of the placebo (348 ± 29 mL) or HCQS tablets (322 ± 42 mL). The administration of the placebo or HCQS did not affect the taste of the milkshake (*p* = 0.19). 

## 4. Discussion

The direct infusion of bitter solutions in the stomach or duodenum has been the subject of investigation in previous studies for its reported action on appetite-regulating gut peptides, appetite-related sensations and food intake via the gut–brain axis [10,11,16,23,27,34]. Given the hunger-suppressive effects reported, bitter agents may prove valuable as an add-on therapy in order to attain weight loss. Still, evidence of their applicability via oral administration in the form of a film-coated “bitter pill” is lacking. Our pilot study found preliminary evidence that the oral ingestion of tablets containing the bitter tastant HCQS (400 mg, Plaquenil^®^) is unlikely to reduce food intake. The oral ingestion of HCQS tablets may be capable of reducing subjective hunger sensations in the fasted state one hour after intake, but this does not translate into a reduction in milkshake intake. The hunger-suppressive action is most likely mediated by a ghrelin-dependent mechanism that does not involve changes in plasma motilin concentrations.

Motilin is a gut peptide that has been, up until now, insufficiently studied for its role in hunger signaling. mainly because of its absence in rodents [35]. Conversely, motilin is well known for its function in stomach motility because of its role in initiating antral phase III contractions of the MMC. Now, it has become more evident that motilin is involved in hunger signaling in humans [22]. The intravenous infusion of motilin or the motilin receptor agonist erythromycin induces antral contractions and hunger sensations in lean volunteers and volunteers with obesity [36,37,38]. In a previous study by Verbeure et al. [10], intragastric infusion with QHCl (10 µmol/kg body weight) tended to suppress hunger sensations, which corresponded with a reduction in motilin plasma concentrations. Therefore, in our current study, we investigated whether HCQS can exert similar effects on hunger sensations and motilin plasma levels. Although HCQS did not show a statistically significant effect on hunger sensations (*p* = 0.10) in this pilot study, a convincing hunger-lowering trend was still observed. This is in agreement with the formerly reported effect of bitter tastants on hunger sensations [11,16,27,39]. 

We previously showed that the intragastric administration of quinine decreases hedonic food intake, as measured with an ad libitum drinking test at the end of each test visit using a rewarding chocolate milkshake [16]. In contrast, in our current study, the volume of chocolate milkshake intake was not significantly reduced by the intake of HCQS tablets, even though there seemed to be a reduction in hunger sensations at the expected time. A limitation of this study is the low statistical power of this variable due to the small sample size. However, our major target was to detect a reduction in motilin plasma concentration, as seen after QHCl infusion [10], which we hypothesize influences hedonic food intake during the fasted state. As reported previously [16], fourteen female volunteers are sufficient to obtain an effect on motilin release with 80% power and α = 0.05. Because these trends were already clear at ten volunteers in a previous study [9], and in our current study, no trend was visible after 10 volunteers, we chose not to increase the sample size. Moreover, in our current study, motilin plasma concentrations did not seem to be affected by chocolate milkshake intake after both the HCQS and placebo treatment. The average volunteer in the study had an intake of 18 g fat (of which 12 g was saturated), 68 g carbohydrates (of which 56 g was sugars) and 14 g proteins. Considering fat to be a motilin-release stimulator and sugar a motilin-release inhibitor, the fat/glucose ratio of the milkshake might neutralize its effect in this volunteer group. This could explain the lack of an effect on motilin plasma concentrations after milkshake intake [40,41]. 

Surprisingly, we found that the oral intake of HCQS did not affect plasma motilin levels, indicating a different mechanism of action compared with intragastric QHCl administration. Instead, our results showed that the intake of HCQS tended to lower plasma acyl-ghrelin concentrations over the course of the study visit with significant differences reached at timepoints consistent with reduced hunger rating. For the interpretation of differences in acyl-ghrelin and motilin values, as evaluated by ANCOVA mixed models, we opted to not correct for multiple comparisons. Because of the exploratory nature of this pilot study, we accept the increased risk of erroneously deducing a significant effect for this variable. However, the observed effect on acyl-ghrelin is in agreement with results reported previously by Iven et al. and Deloose et al., which showed that, besides an effect on motilin, altered acyl-ghrelin plasma concentrations after QHCl administration in the stomach may contribute to lowered hunger sensations [16,27]. Additionally, the timing of the most pronounced effect on acyl-ghrelin (40–50 min after HCQS intake) prompts us to believe that, in our study population, a decrease in acyl-ghrelin plasma concentrations may drive a reduction in hunger ratings caused by HCQS. The 400 mg of HCQS in the Plaquenil tablets is equivalent to 310 mg of base HCQ. The molecular differences between HCQ and QHCl may contribute to the different effects observed on motilin plasma concentrations. Currently, knowledge is lacking on the binding of HCQ to TAS2Rs. HCQ is related to chloroquine, which binds to TAS2R3, 7, 10 and 39, and quinine, which binds to TAS2Rs 4, 7, 10, 14, 39, 40, 43, 44 and 46 [42]. However, HCQ is molecularly different because of its extra hydroxyl group. This modification significantly reduces symptoms associated with its use [43]. It has already been established that TAS2R10 is co-localized with P/D1 cells in vitro, and chloroquine is able to affect ghrelin release from these cell cultures [12], a finding that the current study seems to confirm. It is, therefore, conceivable that the extra hydroxyl group of HCQ does not affect TAS2R affinity, but different molecular actions compared with chloroquine and QHCl cannot be excluded, especially on M-cells. 

One limitation of this study is that we cannot determine the moment that hedonic hunger took over from homeostatic hunger because the volunteers fasted before starting the ad libitum drink test. Ideally, we would offer an ad libitum buffet with rewarding food items and without time restrictions after a standardized breakfast to investigate hedonic food intake after the intake of a bitter pill. However, this would complicate the interpretation of hormone levels, increase the inter-subject variability and make the comparison with previously reported results difficult. Therefore, by only including volunteers who scored liking the chocolate milkshake at 60 mm or more on a 100 mm VAS, our study design ensured the rewarding nature of this beverage and allowed us to pick up changes in hedonic food intake on top of changes in homeostatic food intake in a more reproducible manner. Still, we found no evidence that the reduction in hunger elicited by HCQS translated into a reduction in intake of a rewarding chocolate milkshake. Therefore, we expect limited therapeutic applicability for HCQS-containing bitter pills in weight-loss therapies that focus on dietary intake restriction. Whether pills based on other bitter compounds such as QHCl are able to influence food intake, as seen when QHCl solution was administered directly in the stomach [16], is a subject for further research. In fact, there may be a future for bitter pills in prolonging adherence to fasting regimens independent of food intake [39]. Fasting regimens such as intermittent fasting (IF) have gained popularity and are associated with health benefits such as body weight reduction, increased fat oxidation and decreased fasting insulin levels [44,45,46]. Walker et al. showed that the intake of a hop-containing “bitter pill” in healthy volunteers reduced hunger sensations for at least 4 h in a prolonged fasting state [39]. The potential of HCQS tablets to yield such sustained hunger reduction was not tested in this study. Altogether, these findings steer the development of “bitter pills” more toward appetite-suppressant therapies that suppress hunger during prolonged fasting. However, research into the long-term effects of bitter add-on therapies on subsequent food intake is warranted, especially because it cannot be excluded that long-term bitter compound intake might exert other metabolic effects, change TAS2R expression patterns or shape the gut microbiota, which might affect hunger or body weight through different mechanisms [7].

Finally, we hypothesized a hypoglycemic effect of HCQS through the elevation of insulin levels, as has been reported previously [28,29]. However, this effect could not be proven in our small volunteer group since plasma insulin and blood glucose levels did not differ significantly between the placebo and HCQS treatments. 

In conclusion, our data show that the oral intake of a pill containing HCQS tends to reduce subjective hunger via a ghrelin-dependent mechanism but does not appear to have a hypoglycemic effect, nor does it affect motilin release or feeding behavior in healthy women. 

## Figures and Tables

**Table 1:** Hydroxychloroquine sulfate (400 mg) does not cause any undesirable symptoms. Symptom scores with an absolute value above 10 out of 100 mm VAS were considered in this study. The surface severity (area under the curve) above the cut-off is presented for both the fasted and fed states in the table. Data are presented as IQR (mm).

	Fasted Period		Fed Period	
	IQR (mm)		IQR (mm)	
	Placebo	HCQS	Placebo	HCQS
Belching	0 (0–0)	0 (0–0)	0 (0–0)	0 (0–0)
Nausea	0 (0–0)	0 (0–0)	0 (0–0)	0 (0–0)
Fullness	45 (0–441)	0 (0–86)	1175 (169–1481)	1490 (851–1666)
Cramps	0 (0–0)	0 (0–0)	0 (0–0)	0 (0–0)
Pain	0 (0–0)	0 (0–0)	0 (0–0)	0 (0–0)

## Data Availability

The data presented in this study are available upon request from the corresponding author.
